# On the Chemical Analysis of Vegetables

**Published:** 1801-05

**Authors:** 

**Affiliations:** Berlin


### *On the Chemical Analysis of Vegetables. By Dr.* HERMBSTAEDT, *of Berlin.*

[ Continued from our last, p. 368. ]

### *On the Separation of the Sulphat and Muriat of Kali or Potash.*

THESE neutral salts, if contained in any plant as component parts, are best obtained by incinerating a certain quantity of it, and burning the ashes in an iron pan till they become quite white, and afterwards mixing, and perfectly extracting them with water, and passing the fluid through a filtrum. The lye, which is in this way prepared, must be saturated with distilled vinegar, and entirely evaporated. The remaining dryness is to be digested with alkohol, which takes up all acetated kali, leaving the neutral salts that are formed with the mineral acids undissolved. By dissolving the residuum in water, and chrystallizing it, the salts separate from each other; or in case there existed only one of them in the plant, the separation of this will still better succeed.

### *On the Analysis of the Vegetable Fibre, (Fibra Vegetabilis.)*

After having extracted a plant by every possible dissolvent, and by that means freed it from its component parts, the vegetable fibre remains behind, as a tasteless, ligneous substance, which seldom contains any saline particles, as these are generally extracted with the other parts by the different operations to which the plant has been exposed. All that is to be attended to in the analysis of the vegetable fibre, is to determine the earthy particles, and the carbon which are contained in it; and the analysis is accordingly best performed in the following manner:

1. Put a certain quantity of the vegetable fibre that has been perfectly dried, into a coated glafs retort, to which is joined an adopter or receiver, from the extremity of which iffues a pipe, into which a curved tube is received that is conducted into a bafon with water, for intercepting any gafiform fluid that may be difengaged.

2. Place the retort into a reverberatory furnace, and apply heat at firft gently, but augment it by degrees till the retort becomes red-hot, and no more vapour or gafiform fluid rifes.

3. The quantity of gas that is obtained, may be determined according to cubical inches.* The glafs in which the gas is received, is put with its opening into a veffel full of good lime water, in which it is fuffered to ftand till no more gas is perceived to rife.

4. By fubtracting the quantity which the air in the glafs has been diminifhed, from the whole former mafs, it will be found what quantity of carbonic acid gas was mixed with it, which has been abforbed by the lime water.

5. The remaining gas is generally hydrogen, the quantity of which may be known by the previous diminution.

6. It is feldom the cafe that any fluid is found in the receiver, but when this happens, it is only a little empyreumatic oil, or empyreumatic acid. The refiduum in the retort is the vegetable fibre changed into a black coal.

### *On the Quantity of Carbon contained in the Coal.*

If the weight of the remaining coal be fubtracted from the weight of the vegetable fibre that has been examined, it will be found how much of the heterogeneous particles has been loft. Put now the remaining coal, after having accurately weighed it, into an iron pan, and let it burn with the accefs of air till all black colour difappears. By weighing the remaining white afhes, it will appear what quantity of carbon there was, which after having combined with the oxygen of the atmofpherical air, went off as carbonic acid gas.

### *On the Analysis of the remaining Earth.*

The remaining earth is accurately weighed, and infufed with fix

* For afcertaining the quantity of any gas according to cubical inches, it requires only to be noticed on the glafs, how far it is filled with gas, after which the glafs is filled with an equal quantity of diftilled water, the weight of which is accurately marked. As the Rhenifh duodecimal cubic inch of diftilled water amounts, according to accurate obfervation, to about 290 grains of medicinal weight, at a temperature of 12° Reaumur, the quantity of water is only to be divided with 290, and the quotient will fhow the quantity of the contained gas, according to Rhenifh cubical inches.

ſix times its weight of diſtilled water, and digeſted in a glaſs veſſel, after which the fluid is to be examined by reagency, whether it contains free kali, or any other neutral ſalt. If this be the caſe, the earth ſhould be repeatedly edulcorated, till all ſaline admixture is removed. The remaining earth muſt undergo the following examination:

1. Infuſe it with diſtilled vinegar, this predominates, and all ſoluble parts are thereby diſſolved. What remains undiſſolved is ſiliceous earth, or ſiliceous and aluminous earth mixed with each other.

2. Infuſe therefore this remaining earth with pure diluted ſulphuric acid, and ſuffer it to digeſt. What remains hereby undiſſolved is pure ſiliceous earth, which muſt be waſhed out, dried, and weighed.

3. The ſolution with ſulphuric acid is ſaturated with mild ammonia. If no turbidneſs be occaſioned, no aluminous earth has been preſent; but if it exiſted it will fall to the bottom, and ought then to be edulcorated, dried, and weighed.

4. The ſolution obtained by the acetous acid muſt now be examined with reagency, in order to know what earths are contained in it; a few drops of a ſolution of Glauber's ſalt will, by the enſuing turbidneſs, ſoon diſcover whether any barytes exiſted in it.

5. If barytes was manifeſted in it by this manner, drop ſulphuric acid to the ſolution as long as any precipitation enſues. The precipitate muſt then be waſhed out and dried, and conſiſts of a mixture of ſulphate of barytes (heavy ſpar,) and ſulphat of lime, (ſelenite, gypſum.)

6. Should the earth alſo contain magneſia, this will be found mixed with the gypſum in form of magneſian vitriol, in the water that has been uſed at (5.) This muſt be gently evaporated as long as any ſelenite particles ſeparate from it, which are put to the former (5), and the remaining fluid is precipitated with cauſtic natrum (mineral alkali), and the precipitate edulcorated and dried, which exhibits the pure magneſia.

7. For ſeparating the heavy ſpar and gypſum obtained at (5), it is neceſſary, after having it dried and accurately weighed, to digeſt it well with 600 parts of diſtilled water, by which means the gypſum is diſſolved, whereas the heavy ſpar remains untouched, the weight of which, ſubtracted from the weight of the whole maſs, will determine the weight of the gypſum; and from the known quantitive proportion of the component parts of the heavy ſpar and gypſum, the quantity of barytes and calcareous earth that was contained in the ſame plant, may be determined.

Conclusion. When we intend to examine and analyse any vegetable body here described, it is always necessary, that the results may prove accurate, to employ a new portion of the plant for the examination of each single component part, and to cast up the sum of every single matter that has been found and separated; and in this way it will be only possible to obtain an accurate knowledge of the true elementary mixture of such a plant. The operator should at the same time observe the utmost accuracy in his experiments, persevere in pursuing them, be indefatigable in repeating several times the same experiment, and attend to the utmost cleanliness of the vessels and the reagency, as, otherwise, accurate and certain results cannot be expected.

---

